# Genotypic Diversity of *Streptococcus mutans* in Caries-Free and Caries-Active Preschool Children

**DOI:** 10.1155/2010/824976

**Published:** 2009-11-23

**Authors:** F. J. S. Pieralisi, M. R. Rodrigues, V. G. Segura, S. M. Maciel, F. B. A. Ferreira, J. E. Garcia, R. C. Poli-Frederico

**Affiliations:** ^1^School of Dentistry, University North of Parana, Londrina, 86041-100 Paraná, Brazil; ^2^School of Biology, University Federal of Pernambuco, 55608-680 Vitória de Santo Antão, Brazil

## Abstract

*Aim*. The aim of the present paper was to evaluate the genotypic diversity of *S. mutans* in caries-free and caries-active preschool children in Brazil. *Design*. Twenty-eight preschool children were examined regarding caries experience by the dmft index. DNA from 280 isolates of *S. mutans* was extracted. *S. mutans* evaluated using to the PCR method, with primers for the glucosyltransferase gene. The genetic diversity of *S. mutans* isolates was analyzed by arbitrary primed-PCR (AP-PCR) reactions. The differences between the diversity genotypic and dmft/caries experience were evaluated by *χ*2 test and Spearman's correlation. *Results*. The Spearman correlation test showed a strong association between genotypic diversity and caries experience (*r* = 0.72; *P* < .001). There were more *S. mutans* genotypes in the group of preschool children with dental caries, compared with the caries-free group. Among the children with more than 1 genotype, 13 had dental caries (2 to 5 genotypes) and 4 were caries-free (only 2 genotypes). *Conclusion*. Our results support the previous findings of genetic diversity of *S. mutans* in preschool children being associated with dental caries. The investigation of such populations may be important for directing the development of programs for caries prevention worldwide.

## 1. Introduction

*Streptococcus mutans* is generally considered to be the principal aetiological agent for dental caries [1, 2], which possesses a variety of mechanisms to colonize tooth surfaces. Clinical isolates of *S. mutans* exhibit considerable variations in their genomes or genes [3]. *S. mutans *species, under certain conditions, is numerically significant in cariogenic biofilms and forms biofilms with other organisms in the oral cavity [4] after the eruption and colonization of primary teeth [5]. Furthermore, epidemiologic surveys have confirmed that higher levels of *S. mutans* organisms in children are associated with a higher incidence of decayed, missing, and filled (dmf) teeth [2, 6]. Conversely, it can be found in populations with no caries or with low caries experience [7, 8]. One possible explanation for their presence in subjects with low caries experience is that* S. mutans *virulence factors can differ between populations with contrasting caries prevalence [9]. 

Bowden [10] pointed out the necessity for understanding the clonality patterns of *S. mutans* in the caries-free subjects where it is important to ascertain whether *S. mutans* populations in subjects free of caries exhibit the same clonal diversity of caries-active groups or not [10].

Several studies have showed genetic heterogeneity among *Streptococcus mutans* strains [11–16]; however, the relationship between caries activity and the genetic diversity of *S. mutans* is still controversial. Alaluusua et al. [17] suggested that caries-active children with high sucrose consumption carried greater ribotype diversity of S. *mutans* compared with caries-free children. Napimoga et al. [18] found that caries-active subjects have more genotypes than caries-free subjects. On the other hand, Kreulen et al. [19] showed a negative correlation between caries activity and genotypic diversity.

The aim of the present paper was to evaluate the genotypic diversity of *S. mutans* in caries-free and caries-active preschool children in Brazil.

## 2. Material and Methods

### 2.1. Subjects

Study participants consisted of 28 preschool children aged between 4 and 5 years old from low socioeconomic level families. They had similar lifestyle, dietary, and oral hygiene habits. The subjects were selected from a group of children attending a nursery located in a medium-sized city from Southern Brazil. All of them were from the day nursery, staying in the nursery for 5 days per week, 8 hours per day. During the sample selection, subjects who had any chronic disease and were using antibiotic in the last 3 months were excluded. The aim and details of the experiments were explained, and the informed consent was obtained from parents and guardians prior to the beginning of the research procedures. Experimental procedures were approved by the Ethical Committee of the University of North of Parana School of Dentistry.

### 2.2. Clinical Examination

The children were examined while sitting on a chair under natural light. Diagnosis was visual, using a mouth mirror and cotton rolls to assist visibility and a periodontal probe to remove any plaque or debris when necessary. 

Caries experience was measured by the dmft (decayed, missing, and filled teeth) index, according to the World Health Organization [20]. The caries experience was dichotomized into two groups: caries-free (dmft = 0) and dental caries children (dmft > 0). The clinical examination was performed by the same examiner (F.J.S.P.). The intraexaminer agreement was high (*κ* = 0.92).

### 2.3. Bacterial Strains and DNA Extraction

*Streptococcus mutans* clinical isolates were obtained from Mitis-Salivarius Agar with bacitracin and potassium telurite [21]. About 10 colonies resembling *S. mutans* from each child were transferred to brain heart infusion broth—BHI (Difco, Detroit, USA) and incubated at 37°C for 48 hours in an anaerobic jar. DNA from 280 isolates were extracted by using a simple DNA preparation in which the cells were washed and boiled for 10 minutes with TE buffer (10 mM Tris/HCl, 1 mM EDTA, pH 8.0) modified from Saarela et al. [13] and Welsh and McClelland [22]. The debris were pelleted and the supernatants were stored in a freezer at −20°C until use.

### 2.4. PCR Analyses

Isolates were confirmed for species identity in PCR reactions with primers specific for gtfB, encoding glucosyltransferase 5′ACT ACA CTT TCG GGT GGC TTGG3′ and 5′CAG TAT AAG CGC CAG TTT CATC3′—(Invitrogen) [23], yielding an amplicon of 517 pb for *S. mutans gtfB* gene. Each reaction consisted of 5 *μ*L template DNA, 1 *μ*M of each primer, 200 *μ*M of each dNTP, 5 *μ*L 1x PCR buffer, 1.5 mM MgCl2, and 1 U Taq DNA polymerase (Invitrogen, São Paulo, Brazil) in a total volume of 25 *μ*L. The amplification reaction was performed in 30 cycles as follows: denaturation 95°C for 30 seconds, annealing at 59°C for 30 seconds, and extension at 72°C for 1 minute. One reference strain (ATCC 25175) was used as a positive control of *S. mutans* and distilled water was used as a negative control. Amplification products were analysed electrophoretically in 1% agarose gels using TBE buffer (89 mmol l^−1^ Tris borate, 89 mmol l^−1^ boric acid, 2 mmol l^−1^ EDTA; pH 8), stained with ethidium bromide and observed under UV light. A 100 bp DNA ladder served as molecular-size marker in each gel. All reactions were repeated at least twice.

### 2.5. AP-PCR Typing

Strains identified as *S. mutans* were genotyped. The genetic diversity of *S. mutans* isolates was analyzed by AP-PCR reactions. The sequences of the primers OPA 02 (5′TGCCGAGCTG3′) and OPA 13 (5′CAGCACCCAC3′) were used. The PCR reactions were performed as follows: 1X PCR buffer (200 mmol l^−1^ Tris-HCl pH 8.4; 500 mmol l^−1^ KCl) with 3.5 mM of MgCl_2_, 0.2 mM of each dNTPs, 0.4 mM of primers, 2.5 U of Taq DNA polymerase, and 2.5 *μ*L of DNA sample. The PCR conditions included 35 cycles of denaturation at 94°C for 1 minute, annealing at 36°C for 2 minutes, extension at 72°C for 2 minutes, with initial denaturation at 94°C for 5 minutes, and a final extension at 72°C for 5 minutes. The eletrophoresis was carried out as described previously; however amplification products were analysed in 2% agarose gel. 

Individual AP-PCR amplicons were marked, and the individual bands were analyzed by using the Dice coefficient (>95%) following Mitchell et al. [24]. A dendrogram was constructed using the UPGMA cluster analysis with the aid of Numerical Taxonomy and Multivariate Analysis System (NTSYS) program (Exeter Software, Setauket, NY).

### 2.6. Statistical Analysis

The differences between the genotypic diversity and dmft/caries experience were evaluated by *χ* ^2^ test and the Spearman's coefficient of correlation. Statistical significance was considered to be at *α* < 0.05. The Software Statistical Package for Social Science, v. 11.5 (SSPS, Chicago, IL, USA) was used for the data analysis.

## 3. Results

**Figure 1:**
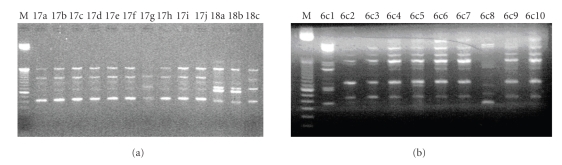
AP-PCR patterns of *S. mutans* isolated from caries-free and dental caries preschool children and detected with OPA-02 (lanes 2–14) and OPA-13 primers (lanes 16–25). Lanes 1 and 15 = size markers 100 bp Ladder (Invitrogen).

A total of 140 isolates of the preschool children with dental caries and 140 isolates of the caries-free preschool children were analyzed by AP-PCR, and 62 different amplitypes were identified. Figure 1 illustrates the AP-PCR patterns performed with OPA-02 and OPA-13, with each of these primers generating a different spectrum of amplicons, indicative of genetic polymorphism.

Characteristics of the children with colonization of *S. mutans* are presented in Table 1. No significant correlation of *S. mutans *was found between genotypic diversity of *S. mutans* and gender. The Spearman correlation test showed a strong association between genotypic diversity and caries experience (*r* = 0.72;  *P* < .001). There were more *S. mutans* genotypes in the group of preschool children with dental caries, compared with the caries-free group. Among the children with more than 1 genotype, 13 had dental caries (2 to 5 genotypes) and 4 were caries-free (only 2 genotypes). 

**Figure 2:**
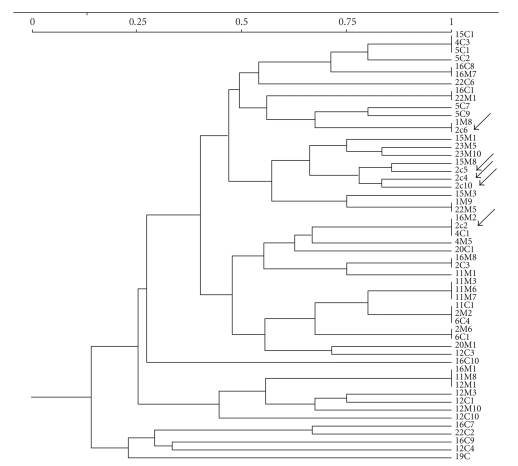
Dendrogram illustrating genotypic diversity between *S. mutans *strains isolated from caries-free and caries children. The Dice coefficient was generated from UPGMA clustering analysis based upon the comparison of the similarity matrices of all *S. mutans *strains type. The arrows indicate the preschool children no. 2c, who showed five distinct genotypes.

Considering the whole population, some of the preschool children harbored just one genotype whereas others exhibited until five genotypes (Figure 2). 

## 4. Discussion

The dental biofilm consists of a complex bacterial community, and the ability of specific strains of *Streptococcus mutans* to compete with other strains may be essential for colonization [25]. Studies of *S. mutans* virulence factors and their correlation with other species are fundamental to understand the role played by colonization of different genotypes in the same individual [26].

The knowledge of genotypic diversity of *S. mutans* may help in the development of new treatment strategies for caries, so as to prevent disease and promote health in addition to standard prevention treatments [26].

Although the findings of Kreulen et al. [19] have demonstrated a negative relationship between caries activity and genotype diversity and the results of Lembo et al. [27] have shown no significant differences in the number of genotypes detected in caries-free and caries-active children, the findings of the present study showed a positive relationship between caries activity and the genetic diversity of *S. mutans*. The preschool children with dental caries have more genotypes than the caries-free children, which is consistent with earlier reports [17, 18, 28, 29]. The existence of several genotypes in the biofilm could merely be a consequence of favorable circumstances for *S. mutans*. Moreover, it is possible that the simultaneous action of different genotypes, with distinct virulence potential, further increases the risk of caries [17].

In studies with young adults, Emanuelsson et al. [29] found a maximum of seven genotypes in subjects who had previously experienced dental caries. Napimoga et al. [18] also found a maximum of eight genotypes in caries-active subjects using AP-PCR. However, it has been observed that children harbor only one to five distinct genotypes of *S. mutans* [3, 11, 15–17, 19, 30]. The results of this research are consistent with previous studies reported in children. It was observed that in the caries-free group, 10 preschool children had only one genotype. On the other hand, in the dental caries group, 13 children had more than one genotype. Of these 13, only 2 harbored five distinct genotypes. This may be attributed to heavy colonization and growth of multiple genotypes in the same oral cavity is likely to be consequences of frequent consumption of fermentable carbohydrates [31]. Different clonal types of *S. mutans* detected within the oral cavity of one subject may exhibit different phenotypic and genetic properties [31]. In addition, the high clonal diversity of *S. mutans* can result in colonization by clones with different virulence attributes [32].

Our results support the previous findings of genetic diversity of *S. mutans* in preschool children being associated with dental caries. The investigation of such populations may be important for directing the development of programs for caries prevention worldwide.

## Figures and Tables

**Table 1:** Distribution of the preschool children with one or more *S. mutans* amplitypes by gender and caries experience (*N* = 28).

	Number of preschool children with	
	1 amplitype	>1 amplitype
*Gender*		
Boys (*n* = 10)	5 (50.0%)	5 (50.0%)
Girls (*n* = 18)	6 (33.3%)	12 (66.7%)
*Caries experienc* e*		
Caries-free preschool children	10 (71.4%)	4 (28.6%)
Preschool children with caries	1 (7.1%)	13 (92.9%)

*Spearman correlation.
